# Role of carnitine and its derivatives in the development and management of type 2 diabetes

**DOI:** 10.1038/s41387-018-0017-1

**Published:** 2018-03-07

**Authors:** Judit Bene, Kinga Hadzsiev, Bela Melegh

**Affiliations:** 1 Department of Medical Genetics, University of Pécs, Medical School, Szigeti 12, Pécs, H-7624 Hungary; 2 Szentágothai Research Centre, University of Pécs, Ifjúság 20, Pécs, H-7624 Hungary

## Abstract

Type 2 diabetes is a highly prevalent chronic metabolic disorder characterized by hyperglycemia and associated with several complications such as retinopathy, hyperlipidemia and polyneuropathy. The dysregulated fatty acid metabolism along with tissue lipid accumulation is generally assumed to be associated in the development of insulin resistance and T2D. Moreover, several studies suggest a central role for oxidative stress in the pathogenesis of the disease. Since L-carnitine (LC) has an indispensable role in lipid metabolism via its involvement in the β-oxidation of long-chain fatty acids and it has antioxidant properties as well, carnitine supplementation may prove to be an effective tool in the management of the clinical course of T2D. In this review we summarize the results from animal and clinical studies demonstrating the effects of supplementation with LC or LC derivatives (acetyl-LC, propionyl-LC) on various metabolic and clinical parameters associated with T2D.

## Introduction

Type 2 diabetes (T2D) is a complex heterogeneous group of metabolic conditions. The hallmark of the disorder is the increased levels of blood glucose due to impaired insulin action and/or insulin secretion. The increasing prevalence of the disorder is spreading intensively throughout the world as a consequence of an aging population and changes in lifestyle. Today, more than 150 million people have been affected worldwide, and this number is estimated to increase to 366 million by the year 2030^1^. There are many complications associated with diabetes, such as atherosclerosis, coronary artery disease, ischemic heart disease, stroke, neuropathy, retinopathy, and nephropathy. An affected carbohydrate and lipid metabolism is clearly established in this disorder. Scientific data support the theory in which the dysregulated fatty acid metabolism along with tissue lipid accumulation are associated with the development of insulin resistance and T2D^2–4^. Since carnitine has a crucial role in fatty acid metabolism, it is likely to be a potential adjuvant in the treatment of T2D. This brief review will focus upon the recent knowledge of carnitine and its derivatives in the development and management of T2D.

### Carnitine homeostasis in humans

Carnitine is a vitamin-like water soluble small molecule featuring a number of essential roles in intermediary metabolism. The primary physiological role is associated with the cellular energy producing processes through the transport of long-chain fatty acids from the cytosol into the mitochondria, where their degradation takes place via β-oxidation. This role is fundamental, since neither the free long chain fatty acids, nor their Coenzyme-A esters can cross the inner mitochondrial membrane on their own; the transport is possible exclusively in carnitine ester form^5^. The acyl moieties are transferred between the Coenzyme-A and the carnitine by the carnitine palmitoyltransferase I and II reversible reactions. The direct transfer of the carnitine esters across the membrane is catalyzed by the carnitine translocase^6^. Beyond its classical physiological role, carnitine has additional crucial functions in the body. Notably, carnitine modifies the acyl-CoA/CoA ratio, which in turn regulates the activity of several mitochondrial enzymes involved in tricarboxylic acid cycle (TCA), fatty acid oxidation, urea cycle and gluconeogenesis^7^. It is involved in energy storage in the form of acetyl carnitine, and modulates the toxicity of partially metabolized acyl groups by facilitating their excretion in carnitine ester form^8^. Furthermore, L-carnitine has been demonstrated to bear anti-inflammatory and antioxidant properties^9–11^ and improves insulin sensitivity, protein nutrition, dyslipidemia, and membrane stability^12^. Due to its pivotal role in intermediary metabolism it is not surprising that plasma and tissue levels of L-carnitine are maintained within a relatively narrow homeostatic range which is controlled by carrier mediated gastrointestinal absorption from dietary sources, endogenous biosynthesis, extensive renal tubular reabsorption, and compartmentalization through carrier-mediated transport between plasma and tissue.

Carnitine is present in the body as free and esterified form (acylcarnitines). Most of the endogenous carnitine pool is distributed between the skeletal and cardiac muscle (approximately 98%) and only less than 1% is located within the plasma. The total carnitine content of human body is in a fairly dynamic state. Carnitine and acylcarnitines migrate among the gastrointestinal tract, the liver, the kidneys and carnitine dependent tissues, such as heart or skeletal muscle. Redistribution between the carnitine and acylcarnitine pools can be observed in the affected tissues after any metabolic change. Kinetics of the carnitine homeostasis and the total carnitine content can vary significantly from tissue to tissue, since carnitine and acylcarnitines cannot directly cross plasma membranes, and carnitine is transported through the membranes by tissue specific transport systems^13^. Dramatic changes can occur in carnitine homeostasis of certain tissues simultaneously with no observable change in others^14^.

Plasma transports only the carnitine and acylcarnitines, therefore it is not surprising in which, their plasmalemmal concentrations are relatively low. In healthy adults, free L-carnitine concentration of plasma is 40–50 μmol/l, that of acetylcarnitine (the most abundant ester) is typically 3–6 μmol/l. Total L-carnitine concentration is approx. 50–60 μmol/l^15^. Since carnitine has no known metabolic function in plasma, changes in plasma carnitine concentrations can be understood only in the relationship with other metabolic or tissue specific information. Majority of the total carnitine content of the body can be found in muscles due to the large mass of the skeletal muscle, and only very small amounts are present in plasma or extracellular compartments. In addition, the concentration of carnitine is much higher in kidney and in liver than in plasma^14^. While total carnitine content of the liver is approximately 500–1000 nmol per gram wet weight, ca. 3000–5000 nmol per gram wet weight can be found in skeletal muscle. Considerable difference in the exchange rates of plasma carnitine with carnitine pools of the two tissues can be observed, as well. There is a rapid exchange between liver carnitine and plasma carnitine and carnitine has a half-life of one to two hours in liver. In contrast, skeletal muscle carnitine does not readily communicate with plasma, and has a half-life of several days^16^. Therefore, changes in carnitine content in liver rapidly appear in plasma, whereas changes in skeletal muscle content may not be as readily apparent in it^17^.

### Carnitine and its derivatives and insulin resistance

Diabetes mellitus is one of the most common chronic metabolic diseases with an underlying absolute or relative insulin deficiency. The main function of insulin is the stimulation of glucose uptake in skeletal muscle for oxidation and storage (as glycogen) and in adipose tissue for synthesis of triacylglycerols. Meanwhile, it inhibits glucose efflux from the liver. In certain pathological conditions, such as T2D, insulin resistance occurs meaning, these tissues show an impaired biological response to either exogenous or endogenous insulin. The measured biological response can be caused by metabolic (changes in carbohydrate, lipid, or protein metabolism) and/or mitogenic processes (alterations in growth, differentiation, DNA synthesis and regulation of gene transcription)^18^. Many theories have been proposed for the molecular mechanism of insulin resistance and its role in glucose and lipid metabolism. One group of theories focuses on the contribution of lipids to the development of insulin resistance.

Several human and animal studies investigated the effect of lipid oversupply on insulin resistance and it has been found in which, via multiple mechanisms, involving the accumulation of intracellular lipids in ectopic tissues (i.e., lipotoxicity), the oversupply of dietary fat leads to insulin resistance^19^. In particular, it was observed in which, within muscle the accumulation of fatty acyl CoA derivatives/metabolites inhibits both insulin signaling and glucose oxidation^4^. Though the exact mechanism is not yet understood, a number of theories have been proposed to assign the signaling event leading to fatty acid induced insulin resistance. One of the most popular hypotheses states that the lipid oversupply results in the accumulation of bioactive lipid metabolites, such as diacylglycerol and fatty acyl-CoA, which activate a serine/threonine kinase cascade, thus, eventually leading to defects in insulin signaling through Ser/Thr phosphorylation of insulin receptor substrate^20^. According to another theory, the long-chain acyl-CoAs are precursors of ceramide, and as such, insulin resistance is ameliorated through ceramide synthesis inhibition^21^. Recent investigations suggest that the lipid oversupply resulted in accumulation of incompletely metabolized fatty acids in the mitochondria causing ‘mitochondrial stress’, leading to insulin resistance^22^.

To explain lipid-induced suppression of muscle glucose disposal, Randle and colleagues proposed the glucose-fatty acid cycle. In regards to this hypothesis acetyl-CoA molecules derived from glucose and lipid substrates compete for entry into the TCA cycle^23^. Recently, Muoio and colleagues suggested an alternative mechanism in which fatty acid oxidation (FAO) rate outpaces that of the TCA cycle, resulting in the accumulation of intermediary metabolites, such as acylcarnitines, which may affect insulin sensitivity^24,25^. Recently, numerous human studies demonstrated in which accumulation of acylcarnitines is associated with insulin resistance, therefore, plasma acylcarnitines have been proposed as biomarkers of insulin resistance^26,27^.

The exact mechanism of the influence of lipid oversupply on insulin resistance is unclear. Since the fundamental biological function of carnitine is its ester-forming capability with organic acids of both exogenous and endogenous origin, it is able to reduce the accumulated acyl CoA derivatives and/or their metabolites via transporting them out from the mitochondria. Therefore, carnitine could be a potential adjuvant in the treatment or prevention of insulin resistance and T2D.

### Impact of L-carnitine supplementation on glucose metabolism

The effect of L-carnitine supplementation on glucose metabolism in humans were widely investigated using a variety of methods (Table 1). Euglycaemic hyperinsulinaemic clamp studies demonstrated that L-carnitine supplementation has an effect on glucose disposal^28–32^. Ferrannini et al. examined healthy young volunteers and discovered intravenous L-carnitine infusion was associated with a significant (17%) stimulation of whole body glucose utilization. Carnitine-induced enhancement of non-oxidative glucose disposal was observed, while net oxidation of glucose was apparently unaffected^28^. In another clamp study, Capaldo et al. investigated the impact of L-carnitine on insulin sensitivity in T2D patients. In this study it was found in which whole body glucose utilization was significantly higher when L-carnitine was infused, currently, there is no additional information available on glucose oxidation under L-carnitine infusion^29^. Mingrone et al.^30^ found that L-carnitine administration resulted in higher whole body glucose uptake and glucose storage in both healthy controls and T2D patients, however, glucose oxidation was increased only in T2D group. In healthy volunteers Stephens et al. investigated the effect of L-carnitine on muscle fuel metabolism. At the end of the clamp study, they demonstrated in which L-carnitine infusion resulted in a 30% increase in muscle glycogen and 40% decrease in muscle lactate content. Furthermore, a 15% increase in skeletal muscle carnitine content was detected as well^31^. In yet another study authored by De Gaetano, healthy human volunteers were subjected to the intravenous glucose tolerance test, coupled with indirect calorimetry after a bolus of glucose plus L-carnitine or a bolus of glucose plus saline. The minimal model, which integrates parameters suitable for the characterization of cellular glucose uptake, sensitivity of pancreatic β-cells to glucose and the kinetics of the delivered insulin, displayed a significant increase in glucose disposal from plasma due to carnitine supplementation. Calorimetry test demonstrated a significant elevation in respiratory quotient, resulting from a significant increase in carbohydrate oxidation rate during carnitine administration^32^. Rahbar et al. investigated the effect of oral L-carnitine administration on fasting plasma glucose, glycosylated hemoglobin (HbA1c) and lipid parameters in T2D patients. Administration of L-carnitine along with pre-existing antidiabetic therapies resulted in significant reduction in fasting plasma glucose level, increased fasting triglyceride levels, whereas HbA1c did not change statistically^33^. In contrast to Rahbar’s findings, Derosa et al. could not find significant effects of oral L-carnitine on fasting plasma glucose in newly diagnosed diabetic patients without diabetic complications^34^. Malaguarnera et al. studied the efficacy of L-carnitine supplementation on plasma glucose and, as well as blood lipid parameters and oxidative stress markers in T2D patients. Although they did not observe significant changes in glucose concentration, the HbA1c level decreased significantly after 12 weeks of treatment. Moreover, a significant decrease was detected in triglyceride, apo A1, apo B-100, and LDL cholesterol, Ox-LDL cholesterol, TBARS (thiobarbituric acid–reactive substances) and conjugated diene concentrations, whereas a significant increase was found in HDL cholesterol concentrations, too^35^. In a pilot study, Molfino et al. demonstrated, that although L-carnitine administration in association with a hypocaloric diet has no effect on fasting glucose concentration, it reduces plasma insulin levels and improves insulin resistance in T2D and IFG patients^36^. Similarly to Molfino, Gonzales-Ortiz et al. reported no significant effect of carnitine supplementation on glucose disposal^37^.

**Table 1:** Effect of carnitine supplementation on glycemic and lipid parameters in human studies with and without diabetic subjects

Subject and characteristics	Study type	Diet	Dosage of carnitine/ day	Adminis-tration	Treatment duration	Carnitine effect on glycemic parameters	Carnitine effect on lipid parameters	Ref
Nine NIDDM patients (7 M/2 F), aged 39–64 y, BMI: 33 oral anti-diabetic medication	EHC	Isocaloric (containing 200 g carbohydrates)	3 mmol LC during EHC	i.v.	3 h	FPG ↔ FINS↔ GUR↑ lactate ↓	FFA↔	^25^
Fifteen T2D patients (8 M/7 F), aged 39.3–55.3 y, 20 healthy volunteers (11 M/9 F)	EHC IC	Weight-maintaining, consisting of 250 g carbohydrates/day for 1 week before the study	0.28 µmol/kg BW/min LC	i.v.	3 h	GUR↑ GOX↑ GSR lactate ↓	FFA↔	^26^
Seven healthy male, mean age:22.4 y, BMI: 26.1	EHC	Standardized, carnitine free during EHC (55% carbohydrates, 35% fat, 10% proteins)	60 mM LC	i.v.	5 h	PDC activity↓ TC_muscle_↑ lactate muscle↓ glycogenmuscle↑	FC_muscle_↑ AC_muscle_ ↔ long-chain acylCoA↑	^27^
Fourteen healthy volunteers (7 M/7 F), aged 33.8±10.9 years, BMI 23.6±2.7	IC	Standard composition (55% carbohydrates, 30% lipids, 15% proteins) ad libitum, with at least 250 g carbohydrates/day	80 mg LC/gglucose	i.v.	One bolus	ISI ↔ GOX↑	FFA↓	^28^
Thirty five T2D patients (22 M/13 F), aged 51.3±3.7 y, disease duration of 12.3±3.4 years, BMI:30, medication with oral anti-diabetic, no insulin or lipid lowering medication	n.m	n.m.	3 g LC	Oral	12 weeks	FPG↓ HbA_1c_↔,	TG↑ LDL-C ↔ HDL-C↔ TC↔ LP(a) ↔ ApoA1↑ ApoB100↑	^29^
Ninety four newly diagnosed T2D patients (47 M/47 F), aged 43–58 y	n.m	Standard composition 1400–1600 kcal/day; 55% carbohydrates, 25% proteins, 20% lipids	2 g LC	Oral	24 weeks	FPG↔ PPG↔ HbA_1c_↔ FINS↔	TG↔ LDL-C↔ HDL-C↔ TC↔ LP(a) ↓ ApoA1↔ ApoB100↔	^30^
Eighty one T2D patients (58 M/23 F) aged 36–62 y	n.m	Standard composition 1400–1600 kcal/day; 55% carbohydrates, 25% proteins, 20% lipids	2 g LC	Oral	12 weeks	FPG↔ HbA_1c_↓	TG↓ Ox-LDL↓ LDL-C↓ HDL-C↑ TC↔ LP(a) ↓ ApoA1↓ ApoB100↓	^31^
Sixteen patients with T2D or IFG (12 M/4 F), no anti-diabetic therapy, aged 49.7–81.7 y, BMI:35.4	n.m	Hypocaloric (1,200 kcal/day F, 1,400 kcal/M; 55% carbohydrates, 25% lipids, 20% proteins)	4 g LC	Oral	2 week	FPG↔ FINS↓ HOMA-IR↓	n.m.	^32^
Twelve T2D patients (6 M/6 F), aged 30–60 years, BMI:30	EHC	Isocaloric diet, containing more than 250 g carbohydrates/day for 3 days before the study	3 g LC	Oral	4 weeks	FPG↔ HbA_1c_↔ GUR↔	TG↔ LDL-C↔ HDL-C↔ TC↔	^33^

*NIDDM* non-insulin-dependent diabetes mellitus, *T2D* type 2 diabetes, *EHC* euglycemic hyperinsulinemic clamp, *IC* indirect calorimetry, *FPG* fasting plasma glucose, *FINS* fasting insulin, *PPG* postprandial plasma glucose, *FFA* free fatty acid, *GUR* whole body glucose uptake rate, *GOX* glucose oxidation rate, *GSR* glucose storage rate, *PDC* pyruvate dehydrogenase complex, *BMI* body mass index (kg/m^2^), *ISI* insulin sensitivity index, *TG* fasting triglyceride, *HDL-C* high-density lipoprotein cholesterol, *LDL-C* low-density lipoprotein cholesterol, *HbA*_*1c*_ hemoglobin A1c, *TC* total cholesterol, *LP(a)*lipoprotein(a), *HOMA-IR* homeostasis model assessment of insulin resistance, = fasting plasma insulin × fasting serum glucose/405, *LC* L-carnitine, *n.m.* not mentioned

Experimental animal studies clearly demonstrate in which carnitine supplementation improves glucose tolerance during insulin- resistant states, such as diabetes or obesity^38^.

Several mechanisms have been suggested in support of the favorable effect of carnitine on glucose metabolism: the enhancing of the mitochondrial oxidation of long chain acyl CoA, the accumulation, of which, would otherwise lead to insulin resistance in muscle and heart; modulating the intramitochondrial acetyl-CoA/CoA ratio and the activity of the pyruvate dehydrogenase complex (PDHC); altering the expression of glycolytic and gluconeogenic enzymes; modifying the expression of genes of the insulin signaling cascade, stimulation of the insulin-like growth factor-1 (IGF-1) axis and IGF-1 signaling cascade^38^.

Although the beneficial effect of L-carnitine supplementation on glucose metabolism was not established in a relative number of human studies, it has improved several T2D related factors, such as, a number of lipid parameters and oxidative stress markers. Therefore, L-carnitine supplementation may have an effect on insulin resistance and possibly be involved in the pathogenesis of T2D. Therefore L-carnitine could be considered to use as an adjuvant in the management of T2D.

### Carnitine and its derivatives in diabetes complications

One of the characteristic hallmarks of T2D is the chronic hyperglycemia. Uncontrolled elevated blood glucose level is supposed to be associated with the development of severe, late diabetic complications, such as neuropathy, retinopathy and nephropathy. Many studies suggest a central role for oxidative stress in the pathogenesis of the disease, therefore, intensive research has been performed in the use of antioxidants as a complementary therapeutic approach to improve the prognosis of diabetic patients with late complications^39^. In addition to its role of lipid metabolism, carnitine possesses antioxidant properties as well, therefore, it has been suggested as an adjunctive in the treatment of diabetes.

Inexplicably, there are a very limited number of studies which effectively investigate the role of L-carnitine levels in the clinical course of diabetes and the development of its late complications. Consequently, the results are reportedly controversial. Poorabbas et al. investigated the free L-carnitine levels in type 2 diabetic women with and without complications. In this study, they found that T2D women with complications displayed almost 25% lower serum free L-carnitine levels than compared with diabetic patients with no complications. However, the free carnitine levels were not significantly different between the groups with retinopathy, hyperlipidemia and polyneuropathy. Furthermore, they did not observe a significant relationship between serum free L-carnitine and blood glucose, lipid profile and systolic and diastolic blood pressure in any of the groups^40^. In another study, Tamamogullari et al. recorded similar results when comparing serum total, free and ester carnitine levels between T2D patients with and without complications. While the levels of total and free carnitines were lower in the patient group with retinopathy, hyperlipidemia and polyneuropathy compared to T2D patients with no complications, there were no significant differences in carnitine levels between the three study groups with different diabetes complications. Moreover, the amounts of esterified carnitine did not show significant differences among all the study groups^41^.

The detected lower levels of carnitine in diabetic patients with diabetic complications encouraged researchers, suggesting that L-carnitine supplementation may likely have therapeutic consequences^18,29^. Although some animal studies^42,43^ demonstrated the beneficial effects of L-carnitine in the management of diabetic complications, in consideration of human studies the effects of L-carnitine in healthy subjects or in T2D patients are yet, controversial^37^. In their study, Liepinsh et al. hypothesized, in which when considering patients with low levels of L-carnitine the occurrence of diabetic complications would have been more frequent, and in these cases, the carnitine supplementation might have a beneficial effect in the prevention of late complications. In this study, they found very similar L-carnitine concentrations in diabetic patient subgroups with or without late diabetic complications, and finally they concluded that L-carnitine concentration is not associated with the prevalence or severity of diabetic complications, such as neuropathy, retinopathy, hypertension, and nephropathy^44^. Briefly, diabetic patients with low levels of L-carnitine did not have increased prevalence of late diabetic complications, and patients with higher average L-carnitine concentrations did not have a decreased prevalence of late diabetic complications.

In addition to L-carnitine its derivatives, acetylcarnitine (ALC) and propionyl-carnitine (PLC), are promising therapeutic agents in the treatment of diabetic complications. ALC has been observed to cross the blood-brain barrier through a sodium-dependent saturable process and improves neuronal energetic and repair mechanisms, while modifying acetylcholine production in the central nervous system^45^. A number of clinical trials were conducted to evaluate the efficacy of ALC in diabetes. De Grandis et al. investigated the efficacy and tolerability of ALC in the treatment of diabetic neuropathy over a 1-year period, focusing on the effects of the treatment on electrophysiological parameters and pain symptoms. They observed in which ALC was well tolerated and improved the neurophysiological parameters and reduced pain^46^. The analysis of two randomized placebo-controlled trials revealed ALC treatment is efficacious in alleviating symptoms, particularly pain, and improves nerve fiber regeneration and vibration perception in patients with established diabetic neuropathy^47^. Clinical trials of ALC administration in type 2 diabetic polyneuropathy have shown beneficial effects on nerve conduction slowing, neuropathic pain, axonal degenerative changes and nerve fiber regeneration^48^. In another study, Giancaterini et al. evaluated the effect of ALC administration on glucose uptake and oxidation rates and they reported the beneficial effects of ALC in patients with T2D mellitus^49^.

PLC has been demonstrated to exert a protective effect in cardiac and endothelial dysfunction, to hinder the progression of atherosclerosis, and to promote some of the cardiometabolic alterations which frequently accompany insulin resistance^50^. Furthermore, PLC protects plasma membranes and reduces vascular-related symptoms in diabetic patients^51^.

### Acylcarnitine analysis in T2D

Several years ago, the investigations of carnitine homeostasis were limited to the analyses of free and total carnitine using classical enzymatic or radioenzymatic assays. Later, the ESI/MS/MS technique was introduced and it enabled the characterization of the alterations in carnitine ester profiles through the highly specific and sensitive determination of various carnitine esters^52^, thus, this tool brought a new perspective to carnitine research. The circulating carnitine ester spectrum can mirror the affected cellular metabolism of amino acids and the short-, medium- and long-chain fatty acids. Due to this diagnostic feature, the mass spectrometric analysis of carnitine profile became a preferred screening test for various inherited metabolic disorders, such as fatty acid oxidation defect, and it has been also used to investigate more common metabolic derangements such as insulin resistance.

Numerous studies utilize mass spectrometric measurements as a tool for analyzing the acylcarnitine profile of T2D, in order to find out the pathogenesis of this metabolic disorder (Table 2). Urine samples taken from diabetes patients were examined using ESI/MS by Möder et al., and as a result they observed that diabetes mellitus patients excrete more long-chain acylcarnitines (C12-C16) than the controls, supposedly caused by a disrupted fatty acid metabolism^53^. Adams and colleagues detected significantly higher levels of plasma acetyl-, medium-chain (C6, C8, C10) and long-chain (C14, C18:1) carnitine esters and lower amount of propionylcarnitine in T2D African-American women. The observed plasma acylcarnitine profiles suggested an incomplete long-chain fatty acid oxidation and altered tricarboxylic acid cycle activity in T2D African-American women^54^. Plasma acylcarnitine profiles were characterized in patients with obesity and T2D during fasting and insulin-stimulated conditions in Mihalik’s study^27^ to find out the site of derangements in FAO and electron transport chain (ETC) activity in obesity and T2D. T2D patients presented increased levels of short- and medium-chain acylcarnitines, both saturated and hydroxyl, as well as C4-dicarboxylcarnitine which showed a correlation with an index of poor glycemic control. Moreover, significantly increased long-chain acylcarnitine concentrations and elevated levels of free carnitine were detected. Insulin infusion resulted in a significant decrease in every acylcarnitine species between carbon lengths from 2 through 18. The observed increased amounts of long-chain acylcarnitine species may likely be the results of increased flux of fatty acids into the mitochondria, whereas the accumulation of many shorter species in T2D suggesting a generalized complex oxidation defect^27^. Dramatically higher amount 8 amino acids (among others leucine/isoleucine and valine) and increased levels of C3 and C5 acylcarnitines in obese versus lean subjects, were found in Newgard’s study^55^. They proposed that elevated levels of branched-chain amino acids are associated with insulin resistance, moreover, the increased branched-chain amino acid catabolic flux may increase gluconeogenesis and may be involved in the development of glucose intolerance via glutamate transamination to alanine^55^. Zhang et al. investigated the serum acylcarnitine profiles in different glucose tolerance states. They observed higher free carnitine and long-chain acylcarnitine levels among the newly diagnosed T2D patients compared to controls, which may suggest different degrees of involvement of dysregulated mitochondrial function and incomplete long-chain fatty acid oxidation pathways in the natural course of T2D^56^.

**Table 2:** Analytical studies of acylcarnitines in humans with and without T2D

Participant characteristics	Sample type	Affected acylcarnitine species	Affected glycemic and lipid parameters	Ref
Twelve lean volunteers (3M/9F), age:47 ± 7, BMI:23.9 ± 1.8 14 obese nondiabetic v. (3M/11F), age:43 ± 7, BMI:34.3 ± 3.1 10 T2D patients (5M/5F), age:45±10, BMI:34.2±2.5	Plasma	T2D vs control: FC↑, C3↑, C4↑, C5↑, C6↑, C10:1↑ (LCAC) C14:1↑, C16↑, C18↑, C18:1↑ (OH) C4OH↑, C5OH↑, C6OH↑, C14OH↑, C16OH↑	FPG↑, HbA_1c_↑	^23^
Eighteen DM patient (6M/12F), 14 controls (6M/8F)	Urine	FC↓, MCAC↑, (C7↑, C8↑, C12↑C14↑) LCAC↑ (C16↑)	n.m.	^49^
Forty four T2D patient (44F), aged 19–87y 12 nondiabetic control (12F), aged 21–69y	Plama	AC↑, AC/FC↑, C2↑, (MCAC) C6↑, C8↑, C10↑, (LCAC)C14↑, C18:1↑ C3↓	FPG↑, HbA_1c_↑	^50^
Seventy four obese volunteers (22M/52F), aged 46–60 y, BMI:36.6 67 lean volunteers (29M/38F), aged 38–60y, BMI:23.2	Serum	C3↑, C5↑, C6↑, C8:1↑	FFA↑, TG↑, HDL-C↓, pyruvate↑, lactate↑	^51^
Twenty patients with normal glucose tolerance (9M/11F), mean age: 48y 20 pre-DM patients (11M/9F), mean age: 51y 21 newly diagnosed T2D patients (8M/13F), mean age: 49y	Serum	T2D vs control: C16↑, C16-OH↑, C20↑, C22↑, C24↑	FPG↑, HbA_1c_↑, FINS↑ IR↑, TC↑	^52^
Forty nine T1D patients (26M/23F), aged 19–64y, BMI: 25.7 38 T2D patients (20M/18F), aged 37–57y, BMI: 29.9 38 MetS patients (19M/19F), aged 34–70y, BMI: 33.8 40 healthy controls (20M/20F), aged 28–71y, BMI: 23.1	Serum	T2D vs control: C2↑, C3↑, C4↑, C5↑, C10:1↓, C12↓, C18:2↓, SCAC↑, MCAC↓, LCAC ↔	n.m.	^53^

*T2D* type 2 diabetes, *DM* diabetes, *T1D* type 1 diabetes, *MetS* metabolic syndrome, *FPG* fasting plasma glucose, *FINS* fasting insulin, *HbA*_*1c*_ hemoglobin A1c, *FFA* free fatty acid, *TG* triglyceride, *HDL-C* high-density lipoprotein cholesterol, *IR* insulin resistance, *TC* total cholesterol, *FC* free carnitine, *AC* total acylcarnitine, *SCAC* short-chain acylcarnitine, *MCAC* medium-chain acylcarnitine, *LCAC* long-chain acylcarnitine, *n.m.* not mentioned

Recently, a study originating from our research group investigated the circulating acylcarnitine profiles in type 1 Diabetes, type 2 Diabetes and metabolic syndrome patients conjecturing in which carnitine homeostasis may likely possess similarities in these metabolic disorders. The carnitine ester profile analysis of adult patients revealed a significantly decreased levels of several medium- and long-chain acylcarnitines in T2D patients compared to the healthy controls, while the levels of free carnitine did not display significant differences. Similar to a previous theory^57^, we inferred in which an inhibited carnitine palmitoyltransferase-1 (CPT1)-mediated entry of free fatty acids into mitochondria may likely be responsible for the observed decreased levels of both long-chain and medium-chain acylcarnitines in our patients. Moreover, sera of T2D patients displayed significantly elevated levels of nearly all short-chain carnitine esters, ranging from C2 to C5 carnitine, thus increased amount of total short-chain esters^58^.

The serum carnitine ester profile consists of metabolites of the amino acid catabolism in addition to those of β-oxidation of fatty acids as well. Odd-chain acylcarnitines originate from amino acid catabolism, whereas the even-chain-length species up to 20 carbons, are predominantly the derivatives of fatty acid β-oxidation intermediates. Whereas C3 derive from isoleucine and valine catabolism, C5 is a byproduct of both leucine and isoleucine catabolism. C4 carnitine ester species can be originated from either fatty acid or amino acid metabolism and C2 produced mostly by carbohydrate catabolism and by β-oxidation including a minor contribution of the degradation of certain amino acids.

The question whether acylcarnitines reflect or inflict insulin resistance was the focus of a recent review^59^. Lipotoxicity is believed to play a crucial role in the induction of insulin resistance and increased attention is turning toward the acylcarnitines via the theory of the role of impairments of fatty acid oxidation in insulin resistance. Acylcarnitines possess distinct functions in the mitochondrial lipid metabolism. It is suggested in which acylcarnitines not only prevent the accumulation of noxious acylCoAs, but also reduce CoA trapping. Additionally, the metabolism of short-chain acylcarnitines and the interaction of acetyl-CoA and acetylcarnitine through carnitine acetyl transferase may regulate the pyruvate dehydrogenase complex, thus have an effect upon glucose oxidation. However, there is an emerging theory on the role of increased, although incomplete, FAO by disproportional regulation of FAO, TCA and respiratory chain in the development of insulin resistance. Schooneman et al. suggest in which acylcarnitines may only, simply reflect the FAO flux and do not play a vital role in the induction of insulin resistance itself^59^. Following this theory, Auger et al. treated differentiated C2C12, primary mouse, and human myotubes with acylcarnitines (C4:0, C14:0, C16:0) and they observed in which the treatment resulted in 20–30% decrease in insulin response at the level of Akt phosphorylation and/or glucose uptake. Eventually, they concluded, incomplete muscle fatty acid β-oxidation causes acylcarnitine accumulation and associated oxidative stress, and these metabolites likely are responsible in the development of muscle insulin resistance^60^. The aim of Liepinsh’s study was to investigate whether there is a beneficial effect of the combination of exercise and long-chain AC decreasing treatment on insulin sensitivity. Their results demonstrated in which long-term acylcarnitine accumulation in the fed state is a feature of T2D. Administration of methyl-GBB (4-ethyl(dimethyl)ammonio-butanoate) resulted in decreased acylcarnitine levels, which, in turn, improved insulin sensitivity and significantly reduced blood glucose and insulin levels in mice with impaired insulin sensitivity and diabetes. Moreover, they experienced that exercise and the combination of methyl-GBB and exercise improved insulin sensitivity in db/db mice. Thus, the reduction of long-chain acylcarnitine content represents an effective strategy towards improving insulin sensitivity^61^.

Today, acylcarnitine profile analysis is extensively used in the investigation of metabolic derangements observable in T2D and several studies demonstrated in which altered AC content is associated with insulin resistance, therefore, pharmacological interventions targeting acylcarnitine accumulation may likely prove to be a promising treatment strategies in the management of T2D.

### Safety concerns of L-carnitine supplementation

In recent years, gut microbiota metabolism of L-carnitine has become a topic of focus in several studies^62–65^. It is reported in which dietary L-carnitine consumption results in TMA (trimethylamine) release via the gut microbiota, which is then converted into TMAO (trimethylamine-N-oxide) by hepatic FMO (flavin monooxygenase). Animal studies suggested that TMAO promote atherosclerosis and increased cardiovascular risk^62,64^, moreover, a significant positive correlation has been found between fasting plasma levels of TMAO and incident major cardiovascular events in a human study^65^.

## Conclusions

Several human and animal studies demonstrated in which L-carnitine supplementation has a beneficial effect on whole body glucose utilization, it improves several lipid parameters or oxidative stress markers as well, moreover, low levels of L-carnitine is associated with various diabetic complications. Furthermore, clinical trials demonstrated that administration of carnitine derivatives, such as, ALC and PLC, improves neurophysiological parameters, reduces pain and reduces vascular-related symptoms in diabetic patients, thus, it could be envisaged as a promising adjuvant in the treatment of diabetes and its complications. However, recent investigations raise the possibility in which L-carnitine-related metabolites exert increased cardio-metabolic risk, therefore further studies will be necessary to effectively evaluate the safety concerns of the administration of L-carnitine.
